# The technological frontier in fall management for older adults with dementia: A scoping review of prediction, detection, and prevention systems

**DOI:** 10.1371/journal.pdig.0001795

**Published:** 2026-11-06

**Authors:** Asmi Khanna, Mansi Bhatia, Sruthi Nunna, Mervin Blair, Tuoyo Awani

**Affiliations:** 1 ClarityPath Research Institute, London, Ontario, Canada; 2 Faculty of Arts & Science, University of Toronto, Toronto, Ontario, Canada; 3 London Health Sciences Centre, Victoria Hospital, London, Ontario, Canada; 4 Schulich School of Medicine and Dentistry, University of Western Ontario, London, Ontario, Canada; University of Cambridge, UNITED KINGDOM OF GREAT BRITAIN AND NORTHERN IRELAND

## Abstract

Falls among older adults represent a major public health concern with substantial clinical, societal, and economic implications. People living with dementia (PlwD) are disproportionately affected, reflecting the combined impact of cognitive, motor, and pharmacological vulnerabilities. Traditional fall risk assessment tools have demonstrated limitations in capturing the complex, dynamic risk profiles of people living with dementia, highlighting the need for technological innovation in prediction, detection, and prevention. Despite advances in gerontechnology, evidence specific to dementia remains limited. To address this gap, a scoping review was conducted following PRISMA-ScR and Joanna Briggs Institute guidelines. Comprehensive searches across Scopus, Medline, Embase, APA PsycINFO, and CINAHL identified thirteen peer-reviewed studies (2015–2025) evaluating technology-based approaches for fall prediction, detection, or prevention among people living with dementia or populations used to model dementia-related functional impairments. Data were extracted and synthesized thematically by technological function. Thirteen studies met inclusion criteria, encompassing pressure-sensitive platforms, technology-enabled preventive interventions, wearable inertial measurement units, and ambient radar/vision-based systems, with the latter two most common. Sample sizes ranged from 3-309 participants with varying dementia severity. Wearables demonstrated strong fall detection performance in controlled laboratory settings but were limited by adherence challenges and reduced ecological validity. Ambient and radar-based systems demonstrated feasibility yet raised privacy and scalability concerns. Pressure-sensitive platforms showed promise for identifying pre-fall risk states but lacked clinical validation. Preventive interventions, including tablet-based exercise programs, demonstrated feasibility and preliminary evidence of reduced fall incidence. These technologies demonstrate promise for dementia care but require further validation, attention to usability, and consideration of ethical implementation before widespread adoption. Future work should emphasize dementia-specific trials, cost-effectiveness, and user-centered co-design to support sustainable implementation.

## Author summary

Falls are a major health concern for older adults with dementia due to the combined effects of cognitive changes, movement difficulties, and medication-related risks. In this study, we examined how technology is being used to predict, detect, and prevent falls in people living with dementia. We reviewed thirteen research studies published between 2015 and 2025 that evaluated different approaches, including wearable sensors, pressure-sensitive platforms, radar-based monitoring, vision-based systems, and tablet-based exercise programs. Wearable devices demonstrated strong accuracy for detecting simulated falls in controlled settings; however, their real-world implementation was limited by challenges related to usability, adherence, and comfort. Ambient technologies, including radar- and vision-based systems, showed potential for continuous monitoring in care environments but raised important considerations related to privacy, scalability, and implementation. Preventive technologies, such as tablet-based exercise programs, demonstrated feasibility and potential benefits for mobility and fall prevention. Overall, this review highlights both the promise and current limitations of technology-based fall management in dementia care. Future research should prioritize larger dementia-specific trials, incorporate user-centered design, and address ethical and economic considerations to support meaningful integration of these tools into everyday care.

## Introduction

### Falls as a geriatric syndrome representing a public health imperative

Falls among older adults are increasingly recognized as a multifactorial geriatric syndrome with profound clinical, societal, and economic consequences [1]. Approximately one-third of community-dwelling adults aged ≥65 years experience at least one fall annually, with 20%–30% sustaining moderate-to-severe injuries such as lacerations, fractures, and traumatic brain injuries [2]. Beyond acute injury, falls often initiate a cascade of functional decline characterized by fear of falling, reduced mobility, deconditioning, loss of independence, diminished quality of life, and premature institutionalization in long-term care facilities [3]. Prolonged periods spent on the floor following a fall, known as the ‘long lie’ phenomenon, further increase the risk of serious complications, hypothermia, dehydration, pressure injuries, pneumonia, hospitalization, and mortality [4].

The societal burden of falls extends beyond their clinical consequences. In the United States alone, annual direct medical expenditures attributable to falls exceed US$80 billion, with costs projected to surpass $101 billion by 2030 [1]. Similar economic pressures have been reported internationally, including an estimated annual cost of £4.4 billion to the United Kingdom’s National Health Service for fall-related fractures [5]. Together, these findings underscore the need for effective strategies to prevent falls and facilitate timely intervention when falls occur.

### Falls in people living with dementia

Although falls are common among older adults, people living with dementia experience a disproportionately greater burden. Compared with cognitively intact older adults, people living with dementia are two-to-three times more likely to fall and nearly four times more likely to experience recurrent falls [6]. Recent meta-analyses estimate an annual fall prevalence of 44.3% among individuals with Alzheimer's disease, with some studies reporting fall rates approaching 80% [6].

This heightened vulnerability reflects the multifactorial pathophysiology of dementia. Progressive neurodegeneration disrupts motor control and gait, resulting in impaired balance, increased postural sway, reduced gait velocity, and mediolateral instability [7]. Concurrent impairments in function, attention, visuospatial processing, and judgment further compromise the ability to recognize hazards and safely navigate complex environments [8]. behavioral and psychological symptoms, including wandering, agitation, and restlessness, increase exposure to hazardous situations [9], while polypharmacy, particularly the use of psychotropic medications and antihypertensive agents, further elevates fall risk through sedation, dizziness, and orthostatic hypotension [10,11].

### Digital technologies for fall management: Rationale for the review

In response to this growing clinical challenge, gerontechnology has produced an expanding range of digital solutions designed to support fall prediction, detection, and prevention [12]. These technologies aim to promote safe and independent aging, improve quality of life, reduce caregiver burden, and alleviate pressure on healthcare systems. However, the populations most likely to benefit from these innovations often face the greatest barriers to their successful implementation. Cognitive impairment, behavioral symptoms, resistance to routine changes, and sensory sensitivities may limit sustained engagement with digital technologies, particularly wearable devices [13,14]. Consequently, technological performance demonstrated under controlled laboratory conditions may not translate into meaningful clinical benefit. Successful implementation therefore depends not only on algorithmic performance, but also on usability, acceptability, ethical deployment, and seamless integration into routine dementia care.

Despite rapid technological advances, the evidence specific to people living with dementia remains fragmented. Previous reviews have examined fall management technologies in older adults more broadly [15] or summarized digital technologies across dementia and mild cognitive impairment populations [16]. However, to our knowledge, no review has comprehensively mapped and synthesized the primary literature specifically evaluating digital technologies for fall prediction, detection, and prevention, tailored for people living with dementia.

Accordingly, this scoping review aims to map and synthesize the current evidence on digital fall management technologies for people living with dementia. Specifically, we sought to (1) characterize the existing evidence across fall prediction, detection, and prevention technologies; (2) identify methodological, technological, and implementation challenges affecting their translation into clinical practice; and (3) highlight emerging opportunities, including artificial intelligence, multimodal sensing, and user-centered design, to inform future research and technology development.

## Methods

### Study design

This scoping review was conducted in accordance with the methodological framework originally proposed by Arksey and O'Malley (2005) and the updated guidance from the Joanna Briggs Institute (JBI) for scoping reviews [17,18]. This review is reported in accordance with the Preferred Reporting Items for Systematic Reviews and Meta-Analyses extension for Scoping Reviews (PRISMA-ScR), and the completed checklist is provided in Table A in S1 Appendix. Given the iterative nature of scoping reviews and the absence of a standard requirement for prospective registration, this review was not preregistered.

Consistent with JBI recommendations, formal methodological quality appraisal or risk-of-bias assessment was not undertaken because the objective was to comprehensively map the breadth, characteristics, and knowledge gaps within the available literature rather than evaluate intervention effectiveness.

### Search strategy

A systematic literature search was conducted across five electronic databases: Scopus, MEDLINE (Ovid), Embase (Ovid), APA PsycINFO (Ovid), and CINAHL (EBSCO). Searches were limited to peer-reviewed studies published between January 2015 and July 2025 to capture contemporary developments in digital fall management technologies.

The search strategy combined controlled vocabulary (e.g., Medical Subject Headings) and free-text keywords using Boolean operators across three conceptual domains: (1) falls and fall risk (e.g., “falls,” “accidental falls,” “fall risk,”); (2) dementia (e.g., “dementia,” “Alzheimer disease,” “cognitive impairment”); and (3) technology-based Interventions (e.g., “technology,” “wearable electronic devices,” “sensors,” “machine learning,” “virtual reality”). A complete search string for MEDLINE via OVID is provided in the **Table B** in S1 Appendix.

To maximize retrieval, reference lists of all included studies and relevant review articles were manually screened for additional eligible publications.

### Eligibility criteria

Inclusion criteria were: (1) studies published in English involving technologies that are specifically designed for older adults, typically defined as individuals aged 65 or older, with a formal diagnosis of dementia; (2) mixed-population studies if data for people living with dementia outcomes were approximated by simulations performed by healthy individuals; (3) studies that evaluated, described, or implemented a specific technology designed for fall prediction, detection, or prevention; (4) at least one fall-related outcome measured, such as a validated fall risk factor (e.g., gait speed, balance), a fall event itself, or an outcome related to a fall prevention intervention (e.g., reduced fall rate, improved balance post-intervention); (5) original research articles, including cohort studies, case-control studies, feasibility or pilot studies, and diagnostic accuracy studies.

Exclusion criteria were: (1) studies designed exclusively for mild cognitively impaired or healthy older adults; (2) interventions limited to non-technological approaches, such as pharmaceutical or behavioral strategies without a technological component; (3) studies classified as gray literature (e.g., conference abstracts, editorials, dissertations).

### Study selection

Search results were imported into Covidence for screening and study management. Title and abstract screening was independently performed by AK and MB, followed by an independent full-text review by the same reviewers. Disagreements were resolved through discussion, with SN acting as a third reviewer when consensus could not be reached.

### Data extraction, charting, and synthesis framework

A standardized data extraction form was developed to extract study characteristics, including publication details, study design, country, participant characteristics (sample size, dementia subtype, and disease severity), technology characteristics (device type, sensor modality, form factor, and intended purpose), algorithmic approach, performance outcomes, and caregiver involvement.

Given the anticipated heterogeneity in study designs, technologies, populations, and outcome measures, quantitative meta-analysis was neither appropriate nor intended. Instead, a narrative thematic synthesis was undertaken to map the existing evidence and identify recurring patterns, methodological limitations, and knowledge gaps.

Studies were organized according to the primary function of the technology within the fall management continuum: (1) fall prediction and risk stratification, referring to technologies designed to identify individuals at increased risk of future falls; (2) fall detection and emergency response, encompassing technologies that identify fall events in real time and initiate an alert or response; and (3) fall prevention and proactive intervention, including technologies intended to reduce fall risk or improve mobility and safety.

Within each category, technologies were further classified according to their sensing modality (e.g., wearable or ambient systems) to facilitate comparison across technological approaches. This framework enabled a structured synthesis of the current evidence while identifying methodological limitations, research gaps, and future opportunities in digital fall management for people living with dementia.

## Results

### Study selection

The databases search identified 207 articles. Following automatic duplicate removal in Covidence, 150 unique records underwent title and abstract screening. After excluding 98 records, 52 full-text articles were assessed for eligibility, of which thirteen studies met the inclusion criteria and were included in the final synthesis. The study selection process is summarized in Fig 1.

**Fig 1:**
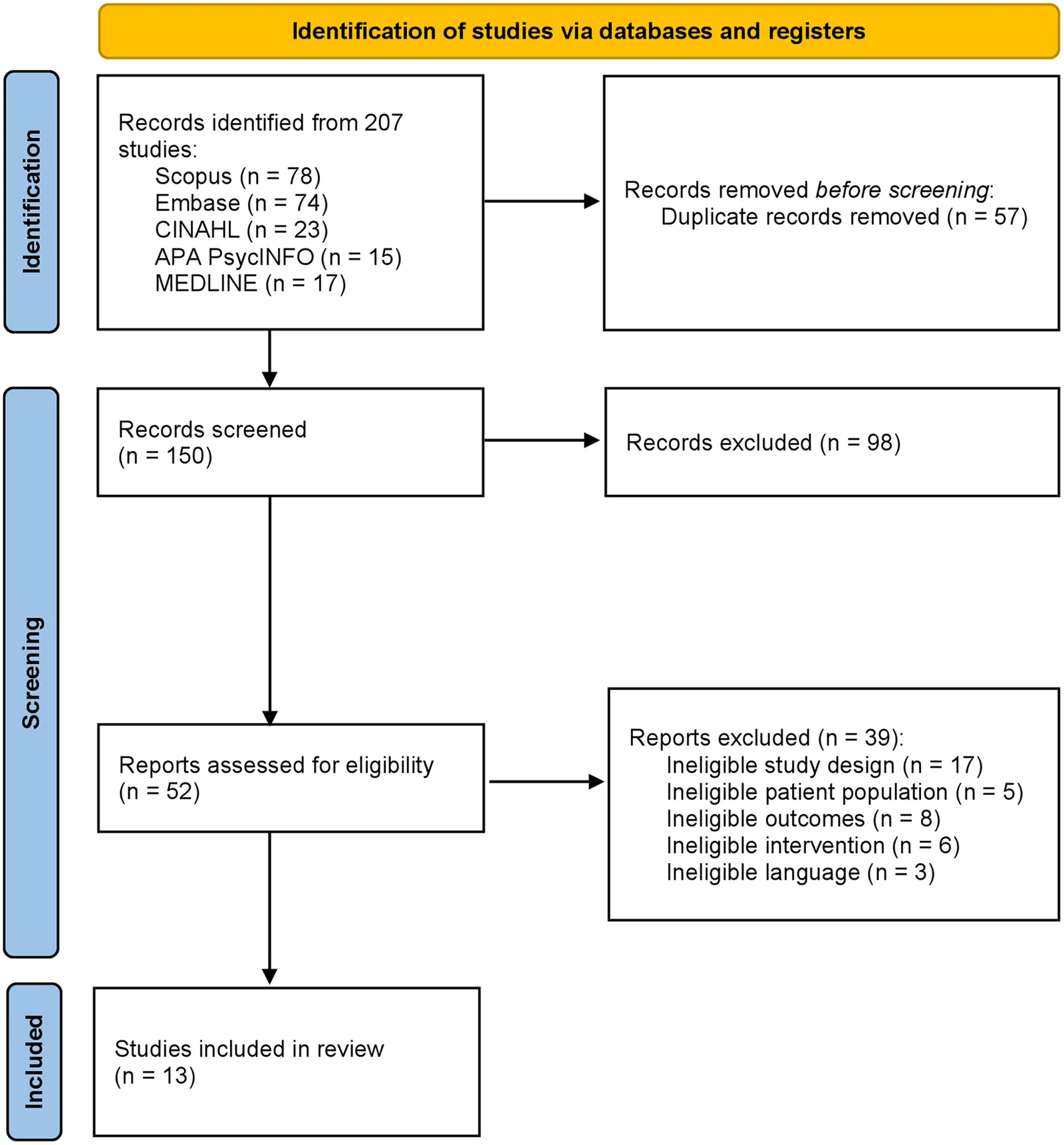
PRISMA-ScR flowchart for study selection and eligibility assessment.

### Characteristics of included studies

The characteristics of the included studies are summarized in Table 1. Thirteen studies published between 2015 and 2025 were included, encompassing a wide range of study designs, clinical settings, and participant populations. Sample size varied considerably, from small feasibility studies involving three healthy volunteers simulating dementia-related movement patterns [19] to larger diagnostic accuracy studies involving more than 30 residents with Alzheimer’s disease [20].

**Table 1:** Study characteristics and population demographics for dementia-targeted falls management technologies.

Index	Author/Year	Country	Settings/ Applied area	Study design	Population	Sample size (*n*)	Groups (*n*)	Sex by group (*n*)	Caregiver involvement
**Wearable Devices**									
1	Szántó and colleagues (2025)	Hungary	Non-clinical (university laboratory and canteen)	Developmental and validation study	The study did not use the target population for testing. Participants were healthy individuals aged between 23 to 26.	17	Fall detection (7) Fingerprint recognition (17)	NR	None (designed to alert caretakers/nurses at a later developmental phase).
2	Mishra and colleagues (2023)	India	Non-clinical	Simulation-based experimental feasibility study	College students who simulated the movements of PlwD.	10	Healthy adults (10)	NR	None (designed to alert caretakers/nurses at a later developmental phase)
3	Ziyad and colleagues (2023)	Saudi Arabia	Non-clinical	Proof-of-concept and algorithm validation study	The study did not recruit any participants. It utilized the open-access IMU Fall Detection Dataset, which contains data collected from 10 healthy young adults.	10	Healthy adults (10)	NR	None (designed to alert caretakers/nurses at a later developmental phase).
4	Schwenk and colleagues (2015)	Germany	Community and hospital	Prospective cohort study	People with a confirmed diagnosis of dementia who had been discharged from a geriatric rehabilitation ward.	77	Fallers (≥1 fall in 3 months) (28) Non-fallers (49)	**Fallers:** 21.4% Male **Non-fallers:** 34.7% Male	None (designed to alert caretakers/nurses at a later developmental phase)
**Ambient Sensors**									
1	Can and colleagues (2024)	Turkey	Assisted living	Pilot prospective observational study	Older residents of a nursing home, specifically from a floor where individuals with the highest risk of falls were clustered. The majority of the study population had dementia. Unspecified dementia, affecting 20 of 26 participants (76.9%).	26	Study group (device installed) (13) Control group (no device) (13)	**Study group:** 1 Male 12 Females **Control group:** 2 Males 11 Females	Nursing and physician staff
2	Adeli and colleagues (2023)	Canada	Assisted living	Prospective longitudinal cohort study	Inpatients in an 18-bed tertiary care Specialized Dementia Unit for older patients with behavioural and psychological symptoms of dementia. Participants had moderate to severe dementia	54	Fallers (25) Non-fallers (29)	**Fallers:** 14 Males 11 Females **Non-fallers:** 16 Males 13 Females	Nursing staff
3	Wright and colleagues (2022)	UK	Inpatient clinic	Retrospective cohort study	PlwD between the ages 57 and 92 years.	NR	Unspecified dementia (NR)	NR	Nursing staff
4	Siedel and colleagues (2020)	Germany	Non-clinical	Feasibility and proof-of-concept study	The fall detection system was tested by three healthy individuals performing fall simulations.	3	Healthy adults (3)	NR	None (designed to alert caretakers/nurses at a later developmental phase)
5	D'Cunha and colleagues (2023)	Australia	Assisted living	Qualitative study	Patients with younger-onset dementia, their family members, and staff.	14	Mild-to-moderate dementia (2) Family members (5) Caregiving staff (7)	**PlwD:** 2 Females **Family members:** 1 Males 4 Female **Staff:** 1 Male 6 Female	Nursing staff, formal caregivers, informal caregivers (family).
6	Yang and colleagues (2015)	Canada	Assisted living	Diagnostic test accuracy study	Older adults living in long term care facilities.	309	Alzheimer’s disease (32)	60.6% Female	Nursing staff
**Pressure-Sensitive Systems**									
1	Wafiq and colleagues (2023)	Bangladesh	Non-clinical	Experimental prototype development and evaluation	Healthy test subjects between the ages 22 to 24 years, who simulated the movements of PlwD.	15	Healthy adults (15)	NR	None (designed to alert caretakers/nurses at a later developmental phase).
2	Chou (2019)	Taiwan	Non-clinical	Developmental study	Healthy test subjects who simulated the movements of PlwD.	NR	NR	NR	Formal caregivers, informal caregivers
**Technology-Enabled Preventive Interventions**									
1	Taylor and colleagues (2020)	Australia	Inpatient clinic	Pilot feasibility study	Patients aged 60 years or older with a diagnosis of dementia or cognitive impairment, who were admitted to a geriatric rehabilitation unit.	43	Intervention Group (21) Control Group (22)	**Intervention Group:** 48% male **Control Group:** 59% male	Informal caregivers, nursing staff

IMU, Inertial Measurement Unit; NR, Not Reported; PlwD, People living with Dementia.

Study populations were heterogenous. Six studies evaluated technologies exclusively using healthy adults or student volunteers performing simulated falls or dementia-related movements [19,21–25], whereas the remaining studies recruited people living with dementia from inpatient, long-term care, assisted living, or community settings [20,26–31].

Dementia subtype and severity were inconsistently reported. Alzheimer’s disease was the most frequently specified diagnosis, whereas several studies included participants with unspecified dementia or younger-onset dementia [30]. Where reported, disease severity ranged from early-stage dementia to moderate and severe dementia in specialized care units [26]. Most clinical studies enrolled fewer than 100 participants.

Sex distribution was also variably reported. Some studies included predominantly female cohorts, for example, Can and colleagues [31] reported over 90% female participants in the intervention group, while others indicated more balanced distributions [26,27]. In contrast, several simulation-based studies did not provide sex data for participants [19,24].

Caregiver involvement was reported primarily in studies involving clinical dementia populations, including nursing staff, formal caregivers, and, less frequently, family caregivers [20,26,27]. In contrast, simulation studies with healthy volunteers did not incorporate caregiver perspectives, with one exception where system design considered caregiver alerts despite their absence from validation procedures [24].

### Characteristics of fall management technologies

The technological characteristics of the included studies are summarized in Table 2. The identified technologies addressed three principal functions within the fall management continuum: fall prediction and risk stratification, real-time fall detection and alerting, and fall prevention.

**Table 2:** Technology and methodological characteristics of dementia-targeted fall management technologies.

Index	Author/ Year	Device brand	Primary purpose	Technology/ sensor type	Reporting time and alerting mechanism	Primary findings	Algorithmic approach	Technology readiness
**Wearable Devices**								
1	Szántó and colleagues (2025)	Custom-built device	Detection, monitoring, location	Combining a microcontroller (STM32 L412KB), communication module (LoRa S76G with GPS), accelerometer/gyroscope (GY-521), PPG heart rate sensor, and a fingerprint reader into a single device.	Real-time, clinician dashboard	The fall detection system achieved 100% accuracy in distinguishing between simulated true falls and false falls (activities like sitting, lying down, and jumping) across 7 participants performing a total of 105 actions (35 true falls, 70 false falls).	NR	Lab prototype
2	Mishra and colleagues (2023)	Custom-built device using an Arduino Nicla SENSE ME board.	Detection, monitoring, alerting	Accelerometer, gyroscope (finger strap)	Real-time, caregiver alert	The optimal model, a Decision Tree classifier, achieved an accuracy (*r*-squared score) of 95% in detecting simulated falls.	ML algorithms: logistic regression, random forest, decision tree, Gaussian Naïve Baye’s, SVM, and K neighbours classifier.	Lab prototype
3	Ziyad and colleagues (2023)	APDM Opal IMU Sensor	Detection, monitoring, alerting	Accelerometer, gyroscope, magnetometer, IMU (ankle, thigh, head, sternum, and waist)	Offline analysis, caregiver alert	The AdaBoost ensemble classifier and the K-Nearest Neighbors (KNN) classifier both achieved 100% accuracy, precision, recall, and F1-score for classifying falls versus activities of daily living on the IMU dataset.	ML AdaBoost	Lab prototype
4	Schwenk and colleagues (2015)	Physilog (BioAGM, CH)	Prediction, monitoring	Accelerometer, gyroscope (chest)	Offline analysis	A prediction model combining a history of previous falls with a sensor-derived measure of 'walking bout average duration' (<15 s) achieved an Area Under the Curve (AUC) of 0.771 (*p* ≤ 0.001). This model predicted falls over a 3-month period with 72% sensitivity and 76% specificity, and an odds ratio of 7.71.	N/A	Deployed, commercial
**Ambient Sensors**								
1	Can and colleagues (2024)	Vayyar	Detection, monitoring, alerting	4D imaging radar camera (wall/ceiling mounted)	Real-time, caregiver alert, clinician dashboard, and alarm	During the 3-month follow-up, there were 2 falls in the control group and 1 fall in the study group. The fall in the study group was detected by the device, which notified staff for a prompt response. The falls in the control group were identified retrospectively by staff with no immediate attention.	Radar-based fall detection algorithm with signal-processing and pattern-recognition methods (proprietary to Vayyar).	Deployed, commercial
2	Adeli and colleagues (2023)	Custom-built device	Prediction, fall risk assessment, monitoring	Microsoft Kinect for Windows v2 as a depth sensor was combined with an RFID system to identify which study participant’s gait was being recorded.	Offline analysis	The best performing ML model predicted falls over the subsequent 4 weeks with an AUROC of 0.762, sensitivity of 0.728, and specificity of 0.732. The model that excluded the ambient gait features only achieved an F1-score of 56.2%, highlighting the significant contribution of the ambient monitoring data. The model achieved its highest AUROC of 77.4% when predicting falls over a 5-week period.	A two-layered MLP as the binary classifier, through SVM, logistic regression, and random forest models were also tested. SMOTE was employed to address imbalanced data.	Pilot tested
3	Wright and colleagues (2022)	Oxehealth	Prediction, monitoring, alerting	RGB camera, infrared (wall/ceiling mounted)	Real-time, caregiver alert	Significant 48% reduction in nighttime falls (*P* < 0.01). Visits from paramedics were reduced by 49% (*P* < 0.2). Accident & Emergency (A&E) admissions were reduced by 68% (*P* < 0.02). Moderate severity falls were reduced by 82%. Enhanced one-to-one observation hours were reduced by 71%.	Unspecified computer vision algorithm	Deployed
4	Siedel and colleagues (2020)	Custom-built Ambient Assisted Living (AAL) system named AUXILIA	Detection, monitoring	RGB camera (omnidirectional camera), infrared, microphone (wall/ceiling mounted), speech recognition component (vicControl DSP 3.2)	Real-time, caregiver alert	The interactive fall detection system achieved a correct detection rate of 85% on a dataset of 553 simulated falls.	MoG, DTW, CLAHE, vicControl DSP 3.2	Lab prototype
5	D'Cunha and colleagues (2023)	Livius	Detection, monitoring, alerting	4D radar sensor (wall/ceiling mounted)	Real-time, caregiver alert	Small-scale dementia care home was a highly successful and satisfactory model for guests, families, and staff because its use of technology supported an overall philosophy of care that empowered staff, promoted a sense of community, and maximized the choice and dignity of risk for residents.	N/A	Deployed, commercial
6	Yang and colleagues (2015)	Custom built device	Monitoring	RGB camera (wall/ceiling mounted)	Offline analysis, clinician dashboard	There was a significant discrepancy between incident reports and video analysis. Agreement was 45.5% for the cause of imbalance, 45.1% for the activity at the time of the fall, and 79.5% for the use of mobility aids.	N/A	Deployed
**Pressure-Sensitive Systems**								
1	Wafiq and colleagues (2023)	Custom-built device	Prediction, monitoring, alerting	Force sensitive resistors combined with an Arduino Mega 2560 microcontroller, an ESP-32 module, and the Blynk IoT platform for remote monitoring and alerts	Real-time, caregiver alert, clinician dashboard	The final optimized system achieved an overall position identification rate of 94.29%. For the specific task of identifying risky positions, it achieved a precision of 0.964, a recall of 0.900, and an F1-Score of 0.931.	Rule-based and threshold-based	Lab prototype
2	Chou (2019)	Custom built device	Detection, monitoring, alerting	Combining Interlink Electronics for the pressure sensors, Arduino for the signal processing module, and RN171 for the Wi-Fi module.	Real-time, caregiver alert, clinician dashboard, and alarm	No numeric results are provided. The study demonstrated through experimental testing that the prototype could successfully distinguish between simulated normal walking (4–6 sensors activated, no alarm) and simulated falls (>7 sensors activated, alarm triggered). It also successfully visualized simulated wandering patterns on the user interface.	Threshold-based: If 7 or more sensors are activated, Arduino then triggers alerts (LED + buzzer) and transmits the information via Wi-Fi to a LabVIEW-based human-machine interface.	Lab prototype
**Technology-Enabled Preventive Interventions**								
1	Taylor and colleagues (2020)	StandingTall (iPad exercise program)	Prevention, monitoring, alerting	Exercise program to improve gait control	N/A	The program demonstrated acceptable feasibility with mean System Usability Scale scores of 68 ± 21 for participants and 69 ± 15 for caregivers, a mean Physical Activity Enjoyment Scale score of 44 ± 8, and an average of 65 exercise minutes in week 12.	The app provides an individually tailored and progressive exercise program, adjusting training intensity based on user-reported Rate of Perceived Exertion ratings after each exercise to ensure the training remains challenging but safe.	Deployed

A&E, Accident & Emergency; AAL, Ambient Assisted Living; AUROC, Area Under the Receiver Operating Characteristic curve; AUC, Area Under the Curve; CLAHE, Contrast Limited Adaptive Histogram Equalization; DSP, Digital Signal Processor; DTW, Dynamic Time Warping; F1-score, F1 (Harmonic Mean of Precision and Recall); GPS, Global Positioning System; IMU, Inertial Measurement Unit; IoT, Internet of Things; KNN, K-Nearest Neighbors; LoRa, Long Range communication; ML, Machine Learning; MLP, Multilayer Perceptron; MoG, Mixture of Gaussians; PACES, Physical Activity Enjoyment Scale; PPG, Photoplethysmography; RPE, Rate of Perceived Exertion; RFID, Radio-Frequency Identification; RGB, Red Green Blue; SMOTE, Synthetic Minority Oversampling Technique; SUS, System Usability Scale; SVM, Support Vector Machine; Wi-Fi, Wireless Fidelity.

Most wearable systems incorporated inertial sensors, including accelerometers, gyroscopes, and inertial measurement units (IMUs) [21,23,24,28]. Ambient monitoring technologies employed radar, depth cameras, red, green, blue (RGB) wavelength cameras, and depth cameras [20,25,26,29–31], while several studies combined multimodal inputs, including radar with real-time pattern recognition [31], and depth cameras integrated with radio-frequency identification (RFID) sensors [26].

Reporting mechanisms involved caregiver notifications, clinician dashboards, or automated alarms; in clinical and assisted living settings, systems were directly integrated with staff workflows to ensure timely emergency response [27,29,31]. In contrast, simulation-based prototypes often restricted outputs to real-time laboratory alerts or offline model validation [19,21,22].

Algorithmic complexity varied substantially. Some studies relied on threshold- or rule-based models [22,25], whereas others implemented machine learning classifiers, including decision trees, random forests, support vector machines (SVMs), AdaBoost, multilayer perceptron, and other ensemble learning methods [21,24,26].

Technology maturity also differed considerably. Several studies evaluated commercially deployed systems within long-term care or assisted-living settings [20,27–31], whereas others focused on laboratory prototypes undergoing preliminary technical validation [19,21–25].

### Technology performance

Performance outcomes varied substantially across studies and were closely associated with study design, participant population, and technology maturity. Studies evaluating prototype systems under simulated conditions consistently reported higher classification performance than those conducted in real-world dementia care settings.

Among simulation-based studies, several machine learning models achieved near-perfect performance. Ziyad and colleagues [24] reported 100% accuracy, precision, recall, and F1-score for fall detection using an AdaBoost ensemble classifier trained on wearable inertial sensor data. Similarly, Szántó and colleagues [23] achieved 100% accuracy when distinguishing between 35 simulated true falls and 70 false fall events using wearable sensors. Mishra and colleagues [21] likewise demonstrated high classification performance, reporting 95% accuracy with a decision tree classifier. Although these findings demonstrate the technical potential of machine learning-based fall detection, they were derived from healthy participants performing simulated falls under controlled laboratory conditions.

Studies involving clinically diagnosed people living with dementia reported more modest but clinically relevant performance. For fall prediction, Adeli and colleagues [26] demonstrated that ambient monitoring of gait characteristics predicted falls occurring within four weeks, achieving an area under the receiver operating characteristic (AUROC) of 0.762, sensitivity of 0.728, and specificity of 0.732. Similarly, Schwenk and colleagues (2015) [32] reported an area under curve (AUC) of 0.771 with 0.72 sensitivity and 0.76 specificity of predicting falls over a 3-month follow-up using wearable sensor-derived gait measures.

Several studies evaluated effectiveness using implementation- or patient-centered outcomes rather than algorithmic accuracy. Wright and colleagues [29] reported a 48% reduction in nighttime falls following implementation a vision-based monitoring system within a dementia care unit, while Taylor and colleagues [27] demonstrated acceptable usability of a tablet-based exercise intervention, with participant usability scores ranging from 68 to 69. These studies highlight that successful implementation may be reflected not only by predictive performance but also by meaningful improvements in safety, usability, and clinical outcomes.

### Technologies for fall detection, emergency response, and prevention

#### Wearable detection devices.

Wearable fall detection systems primarily rely on IMUs, accelerometers, and gyroscopes to identify biomechanical signatures, such as rapid orientation shifts followed by high-impact shocks, which are characteristic of falls. Under simulated conditions, these systems consistently demonstrated excellent classification performance. Mishra and colleagues [21] reported 95% accuracy using a finger-mounted IMU, while Ziyad and colleagues [24] and Szántó and colleagues [23] each achieved 100% accuracy when distinguishing simulated falls from activities of daily living using wearable sensor data.

Most wearable systems, however, were validated using healthy volunteers performing simulated falls rather than people living with dementia, limiting the generalizability of these findings to real-world clinical settings.

#### Ambient and privacy-preserving detection systems.

Ambient monitoring technologies eliminate the need for users to wear a device by continuously monitoring the environment using video, cameras, radar, or other environmental sensors. Yang and colleagues [20] demonstrated the utility of video-based monitoring in long-term care, reporting a modest 45.5% agreement between incident reports and video review regarding the cause of imbalance and 45.1% agreement for the activity being performed at the time of the fall. Similarly, Siedel and colleagues [19] evaluated an interactive ambient monitoring system that correctly detected 85% of 553 simulated falls.

Radar-based systems were also investigated as an alternative ambient sensing approach. In a three-month pilot study, Can and colleagues [31] reported that a commercial four-dimensional imaging radar system successfully detected all falls occurring within the intervention group, enabling immediate caregiver notification following fall events.

#### Technology-enabled fall prevention.

Several studies extended beyond detection to technologies designed to proactively reduce fall risk. Wright and colleagues [29] evaluated the Oxehealth vision-based monitoring platform in dementia care units and reported a 48% reduction in nighttime falls (*p* < 0.01), an 82% reduction in moderate-severity falls, a 68% reduction in emergency department admissions (*p* < 0.02), and a 49% reduction in paramedic visits. Wafiq and colleagues [22] developed a pressure-sensing system integrated into beds and chairs that achieved a 94.3% position identification rate and 96.4% precision for identifying high-risk positions associated with bed falls. Taylor and colleagues [27] evaluated a tablet-based exercise intervention and reported acceptable usability among participants, although the pilot data was not designed to assess reductions in fall incidence.

In summary, detection systems are advancing from wearable IMUs toward ambient, privacy-preserving radar, while prevention strategies increasingly integrate exergames and smart environments. The convergence of these approaches underscores the potential for comprehensive, proactive fall management tailored to the needs of people living with dementia.

## Discussion

This scoping review provides the first comprehensive synthesis of digital technologies developed for fall prediction, detection, and prevention specifically in people living with dementia. Across the thirteen included studies, we identified a rapidly evolving landscape of wearable sensors, ambient monitoring systems, computer vision platforms, radar technologies, and artificial intelligence (AI)-enabled algorithms designed to improve fall management. Collectively, these findings demonstrate considerable technological innovation and suggest that digital health technologies have the potential to support earlier risk identification, facilitate timely intervention following falls, and promote safer, more independent living for people living with dementia.

Despite these promising advances, our findings reveal a substantial gap between technological development and clinical translation. While many systems demonstrated excellent technical performance under controlled laboratory conditions, relatively few were evaluated in real-world dementia care settings, and even fewer examined sustained clinical effectiveness or implementation outcomes. Much of the current evidence remains derived from proof-of-concept studies, small pilot investigations, or simulated falls performed by healthy volunteers, limiting confidence in the generalizability of reported performance metrics to the complex clinical realities of dementia care.

Importantly, this review suggests that successful fall management extends beyond algorithmic accuracy. Although advances in AI and multimodal sensing have improved the ability to classify fall events and identify individuals at elevated risk, successful implementation ultimately depends on technologies that can be integrated into routine care while remaining acceptable to PlwD, caregivers, and healthcare providers. Accordingly, the next stage of innovation should prioritize clinical validation, user-centered design, implementation science, and long-term evaluation alongside continued technological development.

Overall, our findings indicate that the field is transitioning from demonstrating technical feasibility toward addressing the practical challenges of deployment in dementia care. Bridging this translational gap will require future research to move beyond optimizing predictive performance and instead evaluate how digital technologies influence clinically meaningful outcomes, including fall incidence, healthcare utilization, caregiver burden, quality of life, and the ability of people living with dementia to age safely in place.

### Methodological and implementation barriers to clinical translation

#### The “dementia gap”: Limited validation in representative clinical populations.

One of the most significant barriers to the clinical translation of fall management technologies is the persistent “dementia gap” in algorithm development and validation. Six of the thirteen included studies evaluated their technologies using healthy young adults performing simulated falls under controlled laboratory settings [19,21–25]. Although simulated datasets provide an efficient platform for early algorithm development, they fail to capture the complex motor, cognitive, and behavioral characteristics that distinguish falls in dementia.

Falls among people living with dementia arise within a fundamentally different biomechanical and neurological context. Progressive neurodegeneration impairs anticipatory postural adjustments, slows protective reflexes, and disrupts motor coordination, resulting in fall dynamics that differ substantially from the intentional, anticipated falls performed by healthy volunteers [7]. Altered gait initiation, increased stride-to-stride variability, and impaired balance further contribute to movement patterns that are unlikely to be adequately represented in simulated datasets [33]. Consequently, algorithms trained exclusively on healthy participants may identify movement patterns that do not accurately reflect falls occurring in routine dementia care.

This limitation is reflected in the reported performance of many prototype systems. Studies relying on simulated datasets frequently reported classification accuracies approaching 95%–100%, whereas studies conducted in clinical dementia population generally demonstrated more modest performance despite evaluating technologies under substantially more complex real-world conditions. This discrepancy suggests that current performance metrics may overestimate clinical applicability and reinforces the importance of validating technologies using representative dementia populations before widespread implementation.

Beyond population-specific validation, the field would benefit from more rigorous external validation of machine learning models. Most included studies evaluated algorithm performance using data collected from a single institution or experimental environment, with few assessing performances across independent populations, healthcare settings, or sensor platforms. Consequently, reported accuracy may not reflect performance following deployment in routine clinical practice. This challenge is compounded by the relatively small datasets used in many studies together with increasingly complex machine learning models, increasing the risk of overfitting whereby algorithms learn characteristics specific to the training dataset rather than generalizable fall-related movement patterns. Future research should therefore prioritize multicentre prospective validation, standardized benchmarking datasets, and external validation across diverse dementia populations to improve the robustness, reproducibility, and clinical generalizability of these technologies.

#### The human factor: Usability, acceptability, and implementation.

Technical performance alone is insufficient to ensure successful implementation of fall management technologies. Rather, sustained adoption depends on whether technologies can be integrated into the daily lives of people living with dementia. Qualitative research and systematic reviews consistently reveal that devices are often designed without sufficient consideration for the lived experience of aging and dementia [34]. This review highlights that many implementation barriers arise not from algorithmic limitations but from challenges related to usability, acceptability, and long-term adherence.

Wearable technologies exemplify this disconnect between technical capability and real-world implementation. Although inertial sensor systems demonstrated promising predictive performance [21,28], their effectiveness depends on consistent use. Cognitive impairment, memory deficits, behavioral symptoms, sensory sensitivities, and reduced dexterity may all compromise adherence by making devices uncomfortable, difficult to manage or easy to forget [35]. Consequently, technologies that perform well under experimental conditions may experience substantially reduced effectiveness when deployed in routine dementia care.

Although many studies reported high accuracy, sensitivity, and specificity, these metrics alone may not adequately reflect clinical usefulness. False-positive alerts may contribute to alarm fatigue among caregivers and healthcare staff, potentially reducing trust in monitoring systems and diminishing long-term adherence. Conversely, false-negative events may delay emergency response following a fall. Future evaluations should therefore incorporate implementation outcomes such as false alarm rates, caregiver workload, response time, usability, and user satisfaction in addition to conventional algorithmic performance metrics.

Collectively, these findings suggest that the primary barriers to clinical translation are no longer solely technological. Rather, they reflect the need to develop systems that are externally validated, clinically implementable, and acceptable to the people who will ultimately use them. Advancing the field will therefore require greater integration of engineering, clinical science, implementation science, and user-centered design to ensure that technological innovation translates into meaningful improvements in dementia care.

### Evidence gaps limiting clinical implementation

Although many technologies demonstrated promising technical performance, relatively few have generated the level of evidence required for widespread clinical implementation. For a digital health intervention to become standard of care, it must demonstrate technical feasibility and meaningful improvements in patient outcomes, scalability, and economic value under real-world conditions. Our review indicates that the current evidence base falls substantially short of these requirements.

The literature remains dominated by feasibility studies, proof-of-concept investigations, and small observational cohorts, with a notable absence of adequately powered randomized controlled trials evaluating clinically meaningful outcomes. While many studies reported excellent algorithmic performance, few assessed whether these technologies ultimately reduced fall incidence, injury severity, hospitalization, institutionalization, or mortality. Wright and colleagues [29] represents a notable exception, demonstrating a 48% reduction in nighttime falls using a vision-based monitoring system within a dementia care setting. However, studies of this scale and clinical relevance remain uncommon, limiting confidence in the effectiveness of most technologies beyond controlled research environments.

An equally important evidence gap related to economic evaluation. Although some studies have modeled the potential cost-effectiveness of general fall prevention programs like exercise [36], and others have shown digital exercise apps to be cost-saving [37], there is little to no data on the specific return on investment of most new detection or monitoring technologies in dementia care [38]. This limitation represents a critical barrier to implementation, as healthcare organizations increasingly require evidence that digital innovations improve outcomes while remaining financially sustainable. Future research should therefore incorporate formal health economic analyses alongside clinical effectiveness evaluations, considering implementation costs, maintenance requirements, caregiver workload, and downstream savings associated with reduced hospitalizations, delayed institutionalization, and avoided emergency department visits.

More broadly, our findings suggest that fall management technologies are evolving beyond stand-alone detection systems toward integrated digital models of care. Rather than functioning solely as passive fall detectors, future technologies may become components of comprehensive digital care pathways that integrate continuous monitoring, risk prediction, remote rehabilitation, and longitudinal clinical follow-up. Such models have the potential to facilitate earlier intervention, improve continuity of care, and support more proactive, person-centered management of fall risk in people living with dementia.

Collectively, these findings indicate that the principal challenge facing the field is no longer technological innovation but successful implementation. Bridging this gap will require rigorous effectiveness trials, standardized economic evaluations, and implementation science approaches that demonstrate not only whether these technologies work, but how they can be sustainably integrated into routine dementia care.

### Ethical and privacy considerations

The deployment of continuous monitoring technologies within the homes and care environments of people living with dementia raises ethical challenges that extend beyond technical performance. Unlike many other digital health applications, fall management systems frequently operate within highly private spaces and collect continuous behavioral or physiological data from individuals with progressive cognitive impairment. Consequently, successful implementation requires not only technological effectiveness, but also careful consideration of privacy, autonomy, dignity, and public trust.

The central ethical challenge is balancing patient safety with the preservation of privacy and personal autonomy [39]. Technologies capable of providing the richest contextual information, including video- and audio-based monitoring systems, may facilitate more accurate fall detection but are also among the most intrusive, with many users and caregivers expressing concerns regarding surveillance, dignity, and loss of privacy [15]. These concerns have accelerated the development of privacy-preserving alternatives, including four-dimensional imaging radar, depth cameras, thermal imaging, and passive infrared sensors, which enable continuous movement monitoring while minimizing the collection of personally identifiable information. As demonstrated by several studies included in this review, privacy-preserving sensing technologies may offer an important compromise between maintaining patient safety and respecting individual dignity.

Beyond privacy, dementia introduces unique challenges surrounding informed consent and decision-making capacity. Traditional models of one-time informed consent are unlikely to remain sufficient throughout the course of a progressive neurodegenerative disease, as an individual’s capacity to understand ongoing monitoring and exercise autonomous decision-making may change over time [40]. Ethical frameworks must therefore evolve from a static concept of consent to a dynamic process of continual assent, involving ongoing conversations and shared decision-making with the person with dementia, their family, and their legal proxies [39]. Such approaches better reflect the dynamic nature of cognitive decline while preserving autonomy whenever possible.

These considerations are increasingly reflected within international digital health governance frameworks. The World Health Organization’s *Ethics and Governance of Artificial Intelligence for Health* emphasizes transparency, accountability, human oversight, and proportionality as fundamental principles for responsible AI implementation in healthcare [41]. Similarly, the European Union’s *General Data Protection Regulation* highlights data minimization, privacy by design, and explicit governance of sensitive health data, while longstanding frameworks such as the *Belmont Report* continues to provide foundational ethical principles of autonomy, beneficence, and justice as ethical imperatives [42]. Aligning future fall management technologies with these established frameworks may facilitate regulatory approval, increase clinician and caregiver confidence, and promote responsible translation into routine dementia care.

Ultimately, ethical considerations should be viewed as essential design requirements. Technologies that are transparent, privacy-preserving, and developed with sustained engagement from people living with dementia, caregivers, clinicians, and policymakers are likely to achieve greater trust, acceptability, and long-term adoption. Integrating ethical principles throughout the technology development lifecycle will therefore be critical to ensuring that digital innovation enhances safety while preserving dignity and person-centered care.

### Strengths and limitations

The findings of this review should be interpreted within the context of both its strengths and its inherent methodological limitations. As an emerging field, digital fall management for people living with dementia is characterized by rapidly evolving technologies, heterogeneous study designs, and limited high-quality clinical evidence. Consequently, while this review provides a comprehensive synthesis of the current landscape, several considerations are important when interpreting its findings.

Our review has several notable strengths. To our knowledge, this is the first scoping review to comprehensively synthesize primary evidence on technologies for fall prediction, detection, and prevention specifically for people living with dementia. By integrating evidence across engineering, digital health, geriatrics, and dementia care, we provide a broad overview of the current technological landscape while identifying methodological, clinical, ethical, and implementation challenges that may influence future translation into practice. Furthermore, the review was conducted using established Joanna Briggs Institute methodology and reported in accordance with the PRISMA-ScR guidelines, enhancing methodological transparency and reproducibility.

As for limitations, first, the findings should be interpreted in the context of the limited and heterogeneous evidence base currently available. Only 13 studies met the eligibility criteria, encompassing diverse study designs, populations, technologies, outcome measures, and validation approaches. This heterogeneity precluded quantitative synthesis and limited direct comparisons between technologies. Consequently, our findings should be interpreted as mapping the current state of the evidence rather than establishing comparative effectiveness, as would be appropriate for a subsequent systematic review and meta-analysis once the field is more established with a robust number of randomized controlled trials.

Second, many of the included studies were early-stage feasibility or proof-of-concept investigations involving relatively small sample sizes, short follow-up periods, or simulated falls performed by healthy participants rather than people living with dementia. As discussed previously, these methodological limitations reduce the generalizability of reported performance metrics and may overestimate the clinical performance of emerging technologies when implemented in routine dementia care.

### Future directions

Despite current challenges, the future of fall management technology will be shaped by the development of more intelligent, personalized, and clinically meaningful systems. First, future research must address the limited maturity and heterogeneity of the current evidence base. Although this review identified all eligible studies, only thirteen investigations met inclusion criteria, reflecting the early stage of the field. Existing studies vary substantially in design, populations, sensing modalities, algorithms, and outcome measures, with many remaining proof-of-concept studies conducted in controlled environments using simulated falls or small convenience samples. Larger, longitudinal, real-world evaluations are therefore needed to establish generalizability, clinical utility, and comparative effectiveness.

Second, the field should continue shifting from reactive fall detection toward proactive fall prediction and prevention through artificial intelligence and predictive analytics. By integrating longitudinal, multimodal data, including gait, activity patterns, sleep, and physiological measures, AI systems may establish individualized baselines and identify early deviations associated with increased fall risk, enabling timely interventions before falls occur [43]. However, as AI adoption expands, predictive performance must be accompanied by transparency and interpretability. Explainable artificial intelligence (XAI) approaches will be critical for improving clinician trust, supporting regulatory acceptance, and ensuring that predictions meaningfully inform rather than replace clinical decision-making. Beyond fall detection and monitoring, emerging technology-assisted assessment and telemedicine-enabled rehabilitation models in neurological populations may offer opportunities to integrate remote assessment, longitudinal monitoring, and personalized intervention into future dementia care pathways [44–46]. However, dementia-specific validation remains necessary before these approaches can be translated into routine practice.

Third, future technologies should prioritize multimodal sensor integration and comprehensive care platforms. Current evidence remains dominated by single-modality systems, particularly wearable inertial measurement units, despite the potential advantages of combining complementary technologies such as wearables, radar, and vision-based sensors. Advancing sensor fusion approaches, as demonstrated by studies such as Adeli et al. [26], may improve robustness and accuracy in complex real-world environments. Concurrently, the field should move beyond fragmented, single-purpose ‘point solutions’ toward integrated platforms. Instead of requiring a person with dementia and their caregiver to manage a disparate collection of devices for falls, medication, and communication, these functions should be consolidated into unified systems, such as comprehensive smart home networks or multi-functional smartwatches [47]. This approach simplifies the user experience, reduces the overall technology burden, and allows for the creation of more holistic care and support ecosystems.

Fourth, future evaluations must extend beyond technical performance metrics to examine meaningful clinical and patient-centered outcomes. Research should determine whether these technologies improve independence, mobility, quality of life, caregiver burden, and long-term care outcomes among people living with dementia.

Finally, successful implementation will require a shift toward user-centered co-design approaches, involving people living with dementia, caregivers, and healthcare professionals throughout the technology development lifecycle [48]. Collaborative development processes encompassing needs assessment, prototyping, usability testing, and implementation planning are essential to ensure that future innovations are not only technically effective but also acceptable, accessible, and sustainable within dementia care.

## Conclusion

Technology holds immense potential to transform fall management in people living with dementia by enabling objective, continuous, and personalized approaches to risk assessment, detection, and prevention. Emerging innovations, including wearable biosensors, privacy-preserving ambient systems, and interactive virtual reality interventions, demonstrate promising applications. However, real-world evidence remains limited. Current literature is dominated by small-scale feasibility studies, often constrained by methodological heterogeneity, and a persistent dementia validation gap, leaving uncertainty regarding usability, acceptability, clinical benefit, scalability and long-term impact.

Future progress requires a shift beyond proof-of-concept research toward rigorous, longitudinal evaluations in representative dementia populations using standardized outcomes and transparent reporting. Successful translation will depend on human-centered co-design, integration of multimodal and privacy-preserving technologies, and evaluation of meaningful outcomes including falls, injuries, healthcare utilization, caregiver burden, and quality of life. Clinicians, researchers, technologists, and policymakers must work collaboratively to ensure that validated technologies are implemented as part of comprehensive, person-centered dementia care. By aligning technological innovation with clinical evidence, cost-effectiveness, and usability, gerontechnology can meaningfully enhance safety, independence, and dignity for the growing population of people living with dementia.

## Supporting information

S1 Appendix Table of Contents. **Table A.** PRISMA-ScR Checklist. Preferred Reporting Items for Systematic reviews and Meta-Analyses extension for Scoping Reviews (PRISMA-ScR) Checklist. **Table B.** MEDLINE Sample Search Strategy.
(DOCX)

## References

[1] Houry D, Florence C, Baldwin G, Stevens J, McClure R. The CDC injury center’s response to the growing public health problem of falls among older adults. Am J Lifestyle Med. 2015;10(1):74–7. doi: 10.1177/1559827615600137 PMC4681302 26688674

[2] Facts About Falls. U.S. Centers for Disease Control and Prevention [Internet]. 2026 Jan 27. Available from: https://www.cdc.gov/falls/data-research/facts-stats/index.html

[3] Schoene D, Heller C, Aung YN, Sieber CC, Kemmler W, Freiberger E. A systematic review on the influence of fear of falling on quality of life in older people: Is there a role for falls?. Clin Interv Aging. 2019;14:701–19. doi: 10.2147/CIA.S197857 31190764 PMC6514257

[4] Kubitza J, Schneider IT, Reuschenbach B. Concept of the term long lie: A scoping review. Eur Rev Aging Phys Act. 2023;20(1). doi: 10.1186/s11556-023-00326-3 PMC10463813 37644386

[5] Office for Health Improvement & Disparities. Falls: Applying all our health [Internet]. 2022 Feb 25. Available from: https://www.gov.uk/government/publications/falls-applying-all-our-health/falls-applying-all-our-health

[6] Mahmoudzadeh Khalili S, Simpkins C, Yang F. A meta-analysis of fall risk in older adults with Alzheimer’s disease. J Am Med Dir Assoc. 2024;25(5):781-788.e3. doi: 10.1016/j.jamda.2024.01.005 38378160 PMC11065606

[7] Kahya M, Moon S, Ranchet M, Vukas RR, Lyons KE, Pahwa R, et al. Brain activity during dual task gait and balance in aging and age-related neurodegenerative conditions: A systematic review. Exp Gerontol. 2019;128:110756. doi: 10.1016/j.exger.2019.110756 31648005 PMC6876748

[8] Yogev‐Seligmann G, Hausdorff JM, Giladi N. The role of executive function and attention in gait. Movement Disorders. 2007;23(3):329–42. doi: 10.1002/mds.21720 PMC2535903 18058946

[9] Behavioral and psychological symptoms in Alzheimer’s dementia and vascular dementia. In: Handbook of Clinical Neurology [Internet]. Elsevier; 2019 [cited 2026 Jul 17]. p. 5–32. Available from: https://linkinghub.elsevier.com/retrieve/pii/B9780444640123000022 10.1016/B978-0-444-64012-3.00002-2 31727229

[10] Gareri P, Gallelli L, Gareri I, Rania V, Palleria C, De Sarro G. Deprescribing in older poly-treated patients affected with dementia. Geriatrics. 2024;9(2):28. doi: 10.3390/geriatrics9020028 PMC10961769 38525745

[11] Atee M, Stafford A, Whiting D, Lee YP, Morrow C, Nyakaboyi G, et al. Psychotropic polypharmacy in dementia: a retrospective analysis for people with neuropsychiatric symptoms referred to an Australian dementia support service. Drugs Aging. 2025;42(2):165–75. doi: 10.1007/s40266-024-01177-y 39838223 PMC11799070

[12] Pech M, Sauzeon H, Yebda T, Benois-Pineau J, Amieva H. Falls detection and prevention systems in home care for older adults: Myth or reality?. JMIR Aging. 2021;4(4):e29744. doi: 10.2196/29744 PMC8704100 34889755

[13] Larnyo E, Dai B, Larnyo A, Nutakor JA, Ampon-Wireko S, Nkrumah ENK, et al. Impact of actual use behavior of healthcare wearable devices on quality of life: A cross-sectional survey of people with dementia and their caregivers in Ghana. Healthcare (Basel). 2022;10(2):275. doi: 10.3390/healthcare10020275 35206890 PMC8872618

[14] Husebo BS, Heintz HL, Berge LI, Owoyemi P, Rahman AT, Vahia IV. Sensing technology to monitor behavioral and psychological symptoms and to assess treatment response in people with dementia. A systematic review. Front Pharmacol. 2020;10:1699. doi: 10.3389/fphar.2019.01699 32116687 PMC7011129

[15] Gettel CJ, Chen K, Goldberg EM. Dementia care, fall detection, and ambient-assisted living technologies help older adults age in place: A scoping review. J Appl Gerontol. 2021;40(12):1893–902. doi: 10.1177/07334648211005868 33853428 PMC8514579

[16] Eost-Telling C, Yang Y, Norman G, Hall A, Hanratty B, Knapp M, et al. Digital technologies to prevent falls in people living with dementia or mild cognitive impairment: A rapid systematic overview of systematic reviews. Age Ageing. 2024;53(1):afad238. doi: 10.1093/ageing/afad238 38219225 PMC10788098

[17] Arksey H, O’Malley L. Scoping studies: Towards a methodological framework. Int J Soc Res Methodol. 2005;8(1):19–32. doi: 10.1080/1364557032000119616

[18] Peters MDJ, Marnie C, Tricco AC, Pollock D, Munn Z, Alexander L, et al. Updated methodological guidance for the conduct of scoping reviews. JBI Evid Synth. 2020;18(10):2119–26. doi: 10.11124/JBIES-20-00167 33038124

[19] Siedel R, Scheck T, Perez Grassi AC, Seuffert JB, Apitzsch A, Yu J. Contactless interactive fall detection and sleep quality estimation for supporting elderly with incipient dementia. Curr Direct Biomed Eng. 2020;6(3):388–91. doi: 10.1515/cdbme-2020-3100

[20] Yang Y, Feldman F, Leung PM, Scott V, Robinovitch SN. Agreement between video footage and fall incident reports on the circumstances of falls in long-term care. J Am Med Dir Assoc. 2015;16(5):388–94. doi: 10.1016/j.jamda.2014.12.003 25669670

[21] Mishra S, Ngangbam B, Raj S, Pradhan NR. CURA: Real time artificial intelligence and IoT based fall detection systems for patients suffering from dementia. EAI Endorsed Trans Perv Health Tech. 2023;9. doi: 10.4108/eetpht.9.3967

[22] Wafiq MF, Taz M, Nowrin F, Chowdhury AM, Rahim AHMA, Shawon MdMH, et al. An IoT-based bed fall prediction system using force sensitive resistor. In: 2023 IEEE Region 10 Symposium (TENSYMP) [Internet]. Canberra, Australia: IEEE; 2023 [cited 2026 Jul 17]. p. 1–6. Available from: https://ieeexplore.ieee.org/document/10223481/

[23] Szántó M, Dénes-Fazakas L, Noboa E, Kovács L, Borsos D, Eigner G, et al. Developing a health support system to promote care for the elderly. Sensors. 2025;25(2):455. doi: 10.3390/s25020455 PMC11769229 39860825

[24] Ziyad SR, Altulyan M, Alharbi M. SHMAD: A smart health care system to monitor Alzheimer’s disease patients. J Alzheimer’s Dis. 2023;95(4):1545–57. doi: 10.3233/JAD-230402 37718805

[25] Chou H-C. Remote monitoring and control smart floor for detecting falls and wandering patterns in people with dementia. J Intellig Fuzzy Syst. 2018;36(2):1323–31. doi: 10.3233/jifs-169903

[26] Adeli V, Korhani N, Sabo A, Mehdizadeh S, Mansfield A, Flint A, et al. Ambient monitoring of gait and machine learning models for dynamic and short-term falls risk assessment in people with dementia. IEEE J Biomed Health Inform. 2023;27(7):3599–609. doi: 10.1109/JBHI.2023.3267039 37058371

[27] Taylor ME, Close JCT, Lord SR, Kurrle SE, Webster L, Savage R, et al. Pilot feasibility study of a home-based fall prevention exercise program (StandingTall) delivered through a tablet computer (iPad) in older people with dementia. Australas J Ageing. 2020;39(3):e278–87. doi: 10.1111/ajag.12717 31538401

[28] Schwenk M, Hauer K, Zieschang T, Englert S, Mohler J, Najafi B. Sensor-derived physical activity parameters can predict future falls in people with dementia. Gerontology. 2014;60(6):483–92. doi: 10.1159/000363136 PMC4225487 25171300

[29] Wright K, Singh S. Reducing falls in dementia inpatients using vision-based technology. J Patient Saf. 2022;18(3):177–81. doi: 10.1097/PTS.0000000000000882 34347740 PMC9359773

[30] D’Cunha NM, Isbel S, Bail K, Gibson D. “It’s like home” – A small-scale dementia care home and the use of technology: A qualitative study. J Adv Nurs. 2023;79(10):3848–65. doi: 10.1111/jan.15728 37288758

[31] Can B, Tufan A, Karadağ Ş, DurmuŞ NŞ, Topçu M, Aysevinç B, et al. The effectiveness of a fall detection device in older nursing home residents: A pilot study. Psychogeriatrics. 2024;24(4):822–9. doi: 10.1111/psyg.13126 38634167

[32] Schwenk J, Baehrens D, Haupt A, Bildl W, Boudkkazi S, Roeper J, et al. Regional diversity and developmental dynamics of the AMPA-receptor proteome in the mammalian brain. Neuron. 2014;84(1):41–54. 10.1016/j.neuron.2014.08.044 25242221

[33] Allali G, Annweiler C, Blumen HM, Callisaya ML, De Cock A-M, Kressig RW, et al. Gait phenotype from mild cognitive impairment to moderate dementia: Results from the GOOD initiative. Eur J Neurol. 2016;23(3):527–41. doi: 10.1111/ene.12882 26662508 PMC4769662

[34] Wilson S, Ardle RM, Tolley C, Slight S. Usability and acceptability of wearable technology in the early detection of dementia. Alzheimer’s Dementia. 2022;18(S11). doi: 10.1002/alz.062148 36537475

[35] Dino MJ, Thiamwong L, Xie R, Malacas MK, Hernandez R, Balbin PT, et al. Mobile health (mHealth) technologies for fall prevention among older adults in low-middle income countries: bibliometrics, network analysis and integrative review. Front Digit Health. 2025;7:1559570. doi: 10.3389/fdgth.2025.1559570 40225124 PMC11985854

[36] Pinheiro MB, Sherrington C, Howard K, Caldwell P, Tiedemann A, Wang B, et al. Economic evaluations of fall prevention exercise programs: a systematic review. Br J Sports Med. 2022;56(23):1353–65. doi: 10.1136/bjsports-2022-105747 36302631 PMC9685693

[37] Bajraktari S, Sandlund M, Pettersson B, Rosendahl E, Zingmark M. Cost-effectiveness analysis of the digital fall preventive intervention Safe Step among community-dwelling older people aged 70 and older. Eur J Ageing. 2024;21(1):32. doi: 10.1007/s10433-024-00828-8 39455479 PMC11511800

[38] Howard R, Gathercole R, Bradley R, Harper E, Davis L, Pank L, et al. The effectiveness and cost-effectiveness of assistive technology and telecare for independent living in dementia: A randomised controlled trial. Age Ageing. 2021;50(3):882–90. doi: 10.1093/ageing/afaa284 33492349 PMC8099012

[39] Ballegaard SA, Meyer A, Dindler C, Albrechtslund A. Infrastructuring ethical use of surveillance technology in dementia care. CoDesign. 2024;21(1):95–117. doi: 10.1080/15710882.2024.2370287

[40] Gilbert T, Bosquet A, Thomas-Antérion C, Bonnefoy M, Le Saux O. Assessing capacity to consent for research in cognitively impaired older patients. Clin Interv Aging. 2017;12:1553–63. doi: 10.2147/CIA.S141905 29026293 PMC5627738

[41] World Health Organization. Ethics and governance of artificial intelligence for health: WHO guidance [Internet]. 2021. Available from: https://www.who.int/publications/i/item/9789240029200

[42] National Commission for the Protection of Human Subjects of Biomedical and Behavioral Research. The Belmont report: Ethical principles and guidelines for the protection of human subjects of research. U.S. Government Printing Office; 1979. Available from: https://www.hhs.gov/ohrp/regulations-and-policy/belmont-report/index.html 25951677

[43] Rajagopalan R, Litvan I, Jung TP. Fall prediction and prevention systems: Recent trends, challenges, and future research directions. Sensors. 2017;17(11):2509. doi: 10.3390/s17112509 PMC5713074 29104256

[44] Bove F, Di Lazzaro G, Petracca M, Lo Monaco MR, Ricciardi D, Di Caro F, et al. An early feasibility study for neurological devices: The ARCTRAN study. Neurol Sci. 2025;46(10):5221–33. doi: 10.1007/s10072-025-08367-5 40679690 PMC12488755

[45] Giovannini S, Iacovelli C, Brau F, Loreti C, Fusco A, Caliandro P, et al. RObotic-Assisted Rehabilitation for balance and gait in Stroke patients (ROAR-S): Study protocol for a preliminary randomized controlled trial. Trials. 2022;23(1):872. doi: 10.1186/s13063-022-06812-w 36224575 PMC9558956

[46] Castelli L, Iacovelli C, Malizia AM, Loreti C, Biscotti L, Caliandro P, et al. Assessment of fall risk in neurological disorders and technology: Relationship between silver index and gait analysis. Sensors (Basel). 2026;26(3):840. doi: 10.3390/s26030840 41682354 PMC12899951

[47] Cornelius G, Hodgson W, Maguire R, Egan K. Wearable technology, smart home systems, and mobile apps for the self‑management of patient outcomes in dementia care: systematic review. J Med Internet Res. 2025;27:e65385. doi: 10.2196/65385 PMC12411798 40839866

[48] Rocha IC, Arantes M, Moreira A, Vilaça JL, Morais P, Matos D, et al. Monitoring wearable devices for elderly people with dementia: A review. Designs. 2024;8(4):75. doi: 10.3390/designs8040075

